# Data for dynamics analysis of soil dissolved organic matter. Long term amendment effect

**DOI:** 10.1016/j.dib.2019.104665

**Published:** 2019-10-15

**Authors:** Neil Yohan Musadji, Claude Geffroy-Rodier

**Affiliations:** a Institut de Chimie des Milieux et Matériaux de Poitiers (IC2MP), CNRS, Université de Poitiers, France; b Institut National Supérieur d'Agronomie et de Biotechnologies (INSAB), Université des Sciences et Techniques de Masuku (USTM), Gabon

**Keywords:** DOM, Fluorescence excitation-emission matrixes, DOC content, Urban green waste compost

## Abstract

The data presented here are related to the research paper entitled “Spectral characteristics of soil dissolved organic matter: long-term effects of exogenous organic matter on soil organic matter and spatial-temporal changes” (Musadji et al., 2020). Fluorescence Excitation-Emission Matrixes and DOC content of 39 suction cup soil solutions are given for a control and an urban green waste compost amended soil sampled in spring and autumn. Principal component analysis (PCA) was used to identify possible spatial-temporal trends and to emphasize the long term effect of organic amendment on soil organic matter quality.

**Table:** Specifications Table

Subject	Soil science
Specific subject area	One of the subject area focuses on properties of soils in relation to the management and the seasons through Dissolved Organic Matter bulk analysis.
Type of data	Table Figure
How data were acquired	DOC measurements were performed with a Shimadzu Total Organic Carbon Analyzer (TOC- V CPN, Japan). Fluorescence spectra were recorded with a Spex Fluoromax-2 ISA spectrofluorometer (Jobin Yvon, Edison, USA) equipped with a 450 W Xe lamp. All statistical tests were conducted by R Core Team (2019).
Data format	Raw Analyzed
Parameters for data collection	Each plot was equipped with 12 ceramic suction cups (31 mm, SDEC, France) to collect soil solutions disposed in triplicates at 15, 30, 60 or 100 cm depth.
Description of data collection	Based on the corrected absorbance and EEM fluorescence data, general features and three optical indices were used to further describe the compositional characteristics of soil DOM: (1) A_254_, UV absorbance at 254 nm to estimate aromaticity of DOM; (2) Fluorescence index (FI), the ratio of the emission intensity at 450 nm–500 nm at an excitation of 370 nm, used to reflect the relative microbial contribution (>1.9) or terrestrial plant one (<1.4) [2]; (3) Humification index (HIX), the ratio of the peak integrated area under the emission spectra 435–480 nm and under the sum of the emission spectra 300–345 nm and 435–480 nm at an excitation of 254 nm to reflect important complexity and condensation (H/C ratios) of terrigenous contribution (>10) [3]; (4) Biological index (BIX) is an indicator of the relative contribution of the recently microbially produced DOM, calculated as the ratio of emission intensity at 380 nm–430 nm at excitation 310 nm [4].
Data source location	The study was conducted in the Hydrogeological Experimental Site which is a part of the Network of National Hydrogeological Sites (SNO H+) and of the French network of Critical Zone Observatories: Research and Applications (OZCAR) of the University of Poitiers located in the Regional Observatory of Deffend (OR, Mignaloux-Beauvoir, France, 46°23′29.76″ N, 0°24′55.43944 E)
Data accessibility	Data are with this article
Related research article	N.Y Musadji, L. Lemée, L. Caner, G. Porel, P. Poinot, C. Geffroy-Rodier. Spectral characteristics of soil dissolved organic matter: long-term effects of exogenous organic matter and spatial-temporal changes, 2020 Chemosphere, 240, 1–7.

**Table:** 

**Value of the Data** • This data article focuses on main characteristics to follow dissolved organic matter dynamics • This data set provides a large characterization of 78 soil solutions sampled by suction cups that represents a baseline to discern any changes in French soil quality linked to climate change or soil management strategies • The present data will be useful for comparison with other researches and for future studies in order to make correlations between dissolved organic matter and soil organic matter quality and to measure soil treatment long term impact on soil organic matter.

## 1 Data

Bulk and spectral characteristics of autumn and spring soil percolating waters were monitored from 2011 to 2013 through 1 m depth for control and amended soils [1].

**Fig. 1:**
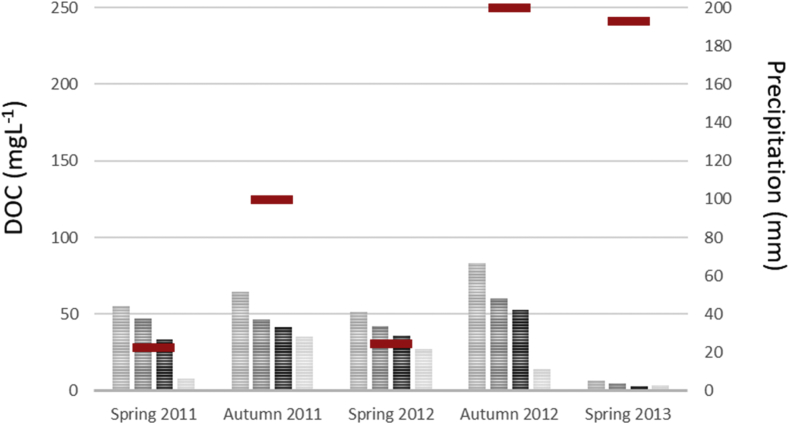
DOC content in grey at 15, 30, 60–100 cm depth (mgL^−1^) and 2 months cumulative precipitation in red (mm) before samplings in autumn and spring.

**Fig. 2:**
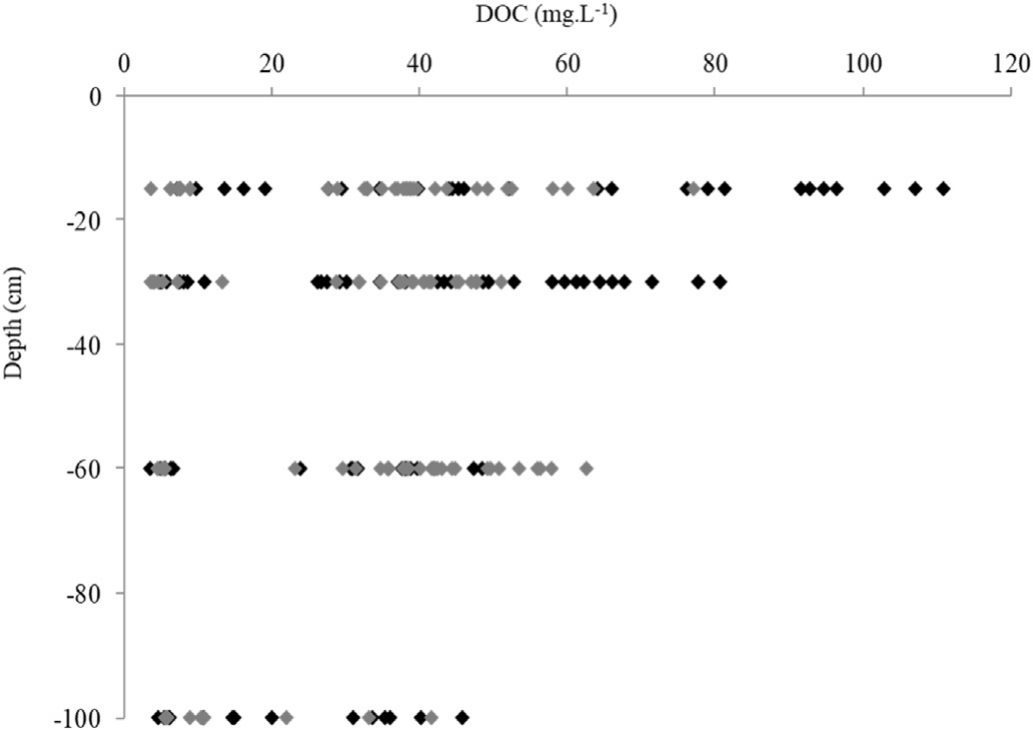
Autumn 2012 DOC content at 15, 30, 60–100 cm depth for control (grey) and amended soil (black).

**Fig. 3:**
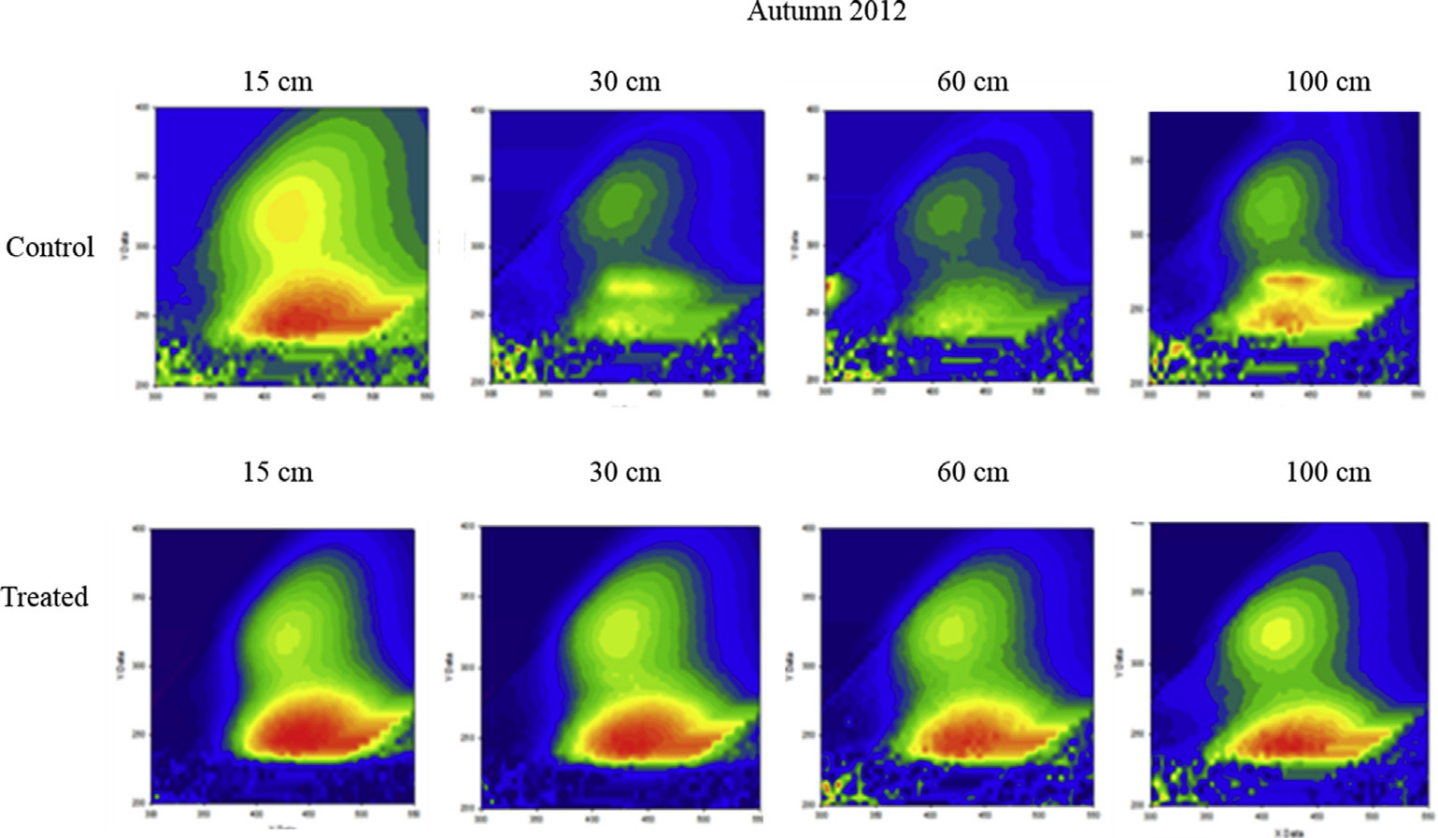
EEMF matrixes of DOM in autumn 2012 for amended and control soil solutions where x-axis and y-axis represent respectively the scan range of emission and excitation wavelengths. Colors show the relative fluorescence intensity of DOM. Letters indicate the main fluorophores (see Table 1).

**Fig. 4:**
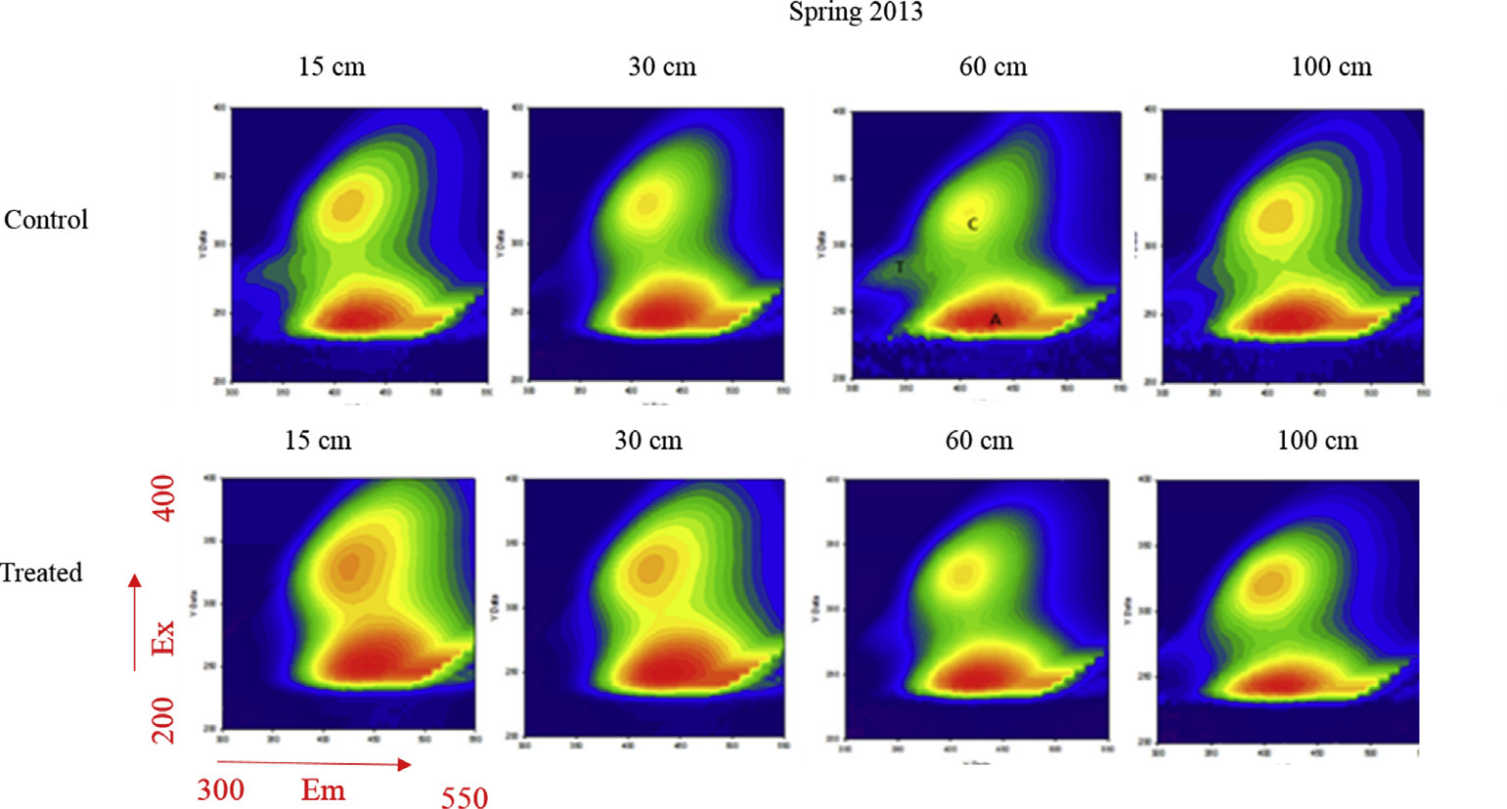
EEMF matrixes of DOM in spring 2013 for amended and control soil solutions where x-axis and y-axis represent respectively the scan range of emission and excitation wavelengths. Colors show the relative fluorescence intensity of DOM. Letters indicate the main fluorophores (see Table 1).

**Fig. 5:**
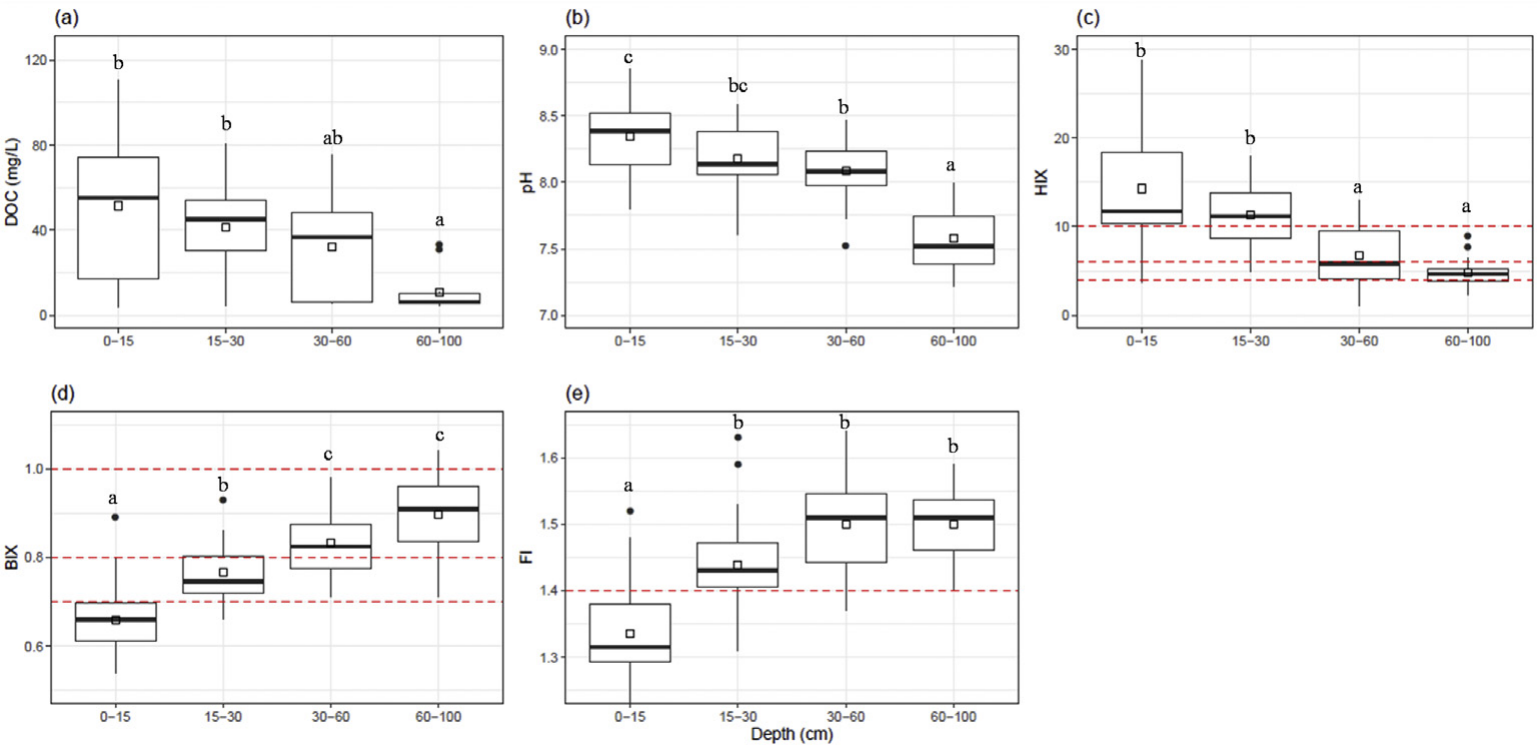
DOC (mg.L^−1^), pH and fluorescent proxies (HIX, BIX and FI) variation of soil solutions along of depth (15, 30, 60 and 100 cm). Boxes plots indicate 25th and 75th percentiles, solid lines within boxes refer to median values, inbox points represent the mean value and the close circles represent the outliers values. The subscripts letters after the data indicate the similarity and the differences. The same letter indicates that the mean values are similar within a group and between groups. Different subscripts represent significant difference between compared groups at p-value < 0.05. The red dotted lines represent the different fluorescent proxies' scales (see Specifications Table).

Mean values of amended soil Dissolved Organic Matter carbon content and the 2 months cumulative precipitation occurred before sampling are reported from 2011 to 2013 (Fig. 1). Dissolved Organic Matter carbon content (Fig. 2) and representative EEM fluorescence spectra (Fig. 3) are given through the depth profile for control and amended soil solutions sampled in autumn 2012. EEM fluorescence spectra of spring 2013 are shown in Fig. 4. Absorbance characteristics of spring 2013 and autumn 2012 samples are given in Table 1. The identified components contained humic-like substances (C/α) and recent materials/fulvic acid-like substances (A/α′) located at λ_ex_/λ_Em_ 330–350/420-440 nm and 240–260/420-480 nm respectively. Protein-like substances (δ/Τ, γ/B) were detected at λ_ex_/λ_Em_ 270–280/300–340 nm. Control soil fulvic acids' characteristics are given as fulvic acid-like substances reference in Table 2. Mean pH, Dissolved Organic Matter content and fluorescent proxies (HIX, BIX FI) are reported in function of depths, seasons and soil treatments (Fig. 5 and Table 2).

**Table 1:** Mean values Absorbance characteristics of fluorescent components of DOM.

Samples & seasons	Depth (cm)	n	A (α′)		C(α)		T(δ)	
Field			λ_ex_	λ_em_	λ_ex_	λ_em_	λ_ex_	λ_em_
AC	0–15	9	246 ± 3^a^	435 ± 8^a^	330 ± 1^a^	422 ± 3^ab^	275 ± 5^ab^	326 ± 20^bc^
	15–30	7	243 ± 4^a^	428 ± 18^a^	332 ± 2^a^	421 ± 2^a^	275 ± 5^ab^	318 ± 20^ac^
	30–60	6	240 ± 3^a^	420 ± 10^a^	330 ± 2^a^	420 ± 0^a^	273 ± 5^ab^	308 ± 16^ab^
	60–100	3	240 ± 5^a^	416 ± 10^a^	330 ± 0^a^	420 ± 0^ab^	278 ± 3^ab^	338 ± 2^bc^
AT	0–15	9	246 ± 2^a^	438 ± 9^a^	330± 0^a^	427 ± 4^ab^	277 ± 4^b^	331 ± 17^bc^
	15–30	8	245 ± 2^a^	431 ± 11^a^	330 ± 1^a^	421 ± 2^a^	275 ± 5^ab^	326 ± 19^ac^
	30–60	6	244 ± 2^a^	425 ± 8^a^	330 ± 0^a^	420 ± 0^a^	270 ± 0^a^	300 ± 0^a^
	60–100	4	243 ± 2^a^	426 ± 4^a^	330 ± 0^a^	420 ± 0.^a^	280 ± 0^b^	340 ± 0^bc^
SC	0–15	3	246 ± 3^a^	423 ± 10^a^	331 ± 3^ab^	421 ± 3^ab^	280 ± 0^b^	340 ± 0^bc^
	15–30	2	245 ± 2^a^	420 ± 0^a^	330 ± 0^ab^	420 ± 0^ab^	280 ± 0^ab^	340 ± 0^ac^
	30–60	3	243 ± 3^a^	426 ± 3^a^	330 ± 0^a^	420 ± 0^ab^	280 ± 0^b^	338 ± 3^bc^
	60–100	4	247 ± 8^a^	416 ± 6^a^	330 ± 0^a^	420 ± 0^a^	280 ± 0^b^	335 ± 7^bc^
ST	0–15	5	248 ± 3^a^	436 ± 5^a^	330 ± 0^a^	424 ± 2^ab^	280 ± 0^b^	340 ± 0^c^
	15–30	3	248 ± 3^a^	431 ± 3^a^	330 ± 0^a^	420 ± 0^ab^	280± 0^b^	340 ± 0^bc^
	30–60	3	245 ± 0^a^	426 ± 3^a^	330 ± 0^a^	420 ± 0^ab^	280 ± 0^b^	340 ± 0^bc^
	60–100	3	248 ± 10^a^	433 ± 23^a^	343 ± 23^b^	433 ± 23^b^	278 ± 3^ab^	338 ± 3^bc^

Notes: AC- control in autumn, AT-treated in autumn, SC- control in spring, ST-treated in spring. The subscripts letters after the data indicate the similarity and the differences. The same alphabet indicates that the compared group are similar. Different subscripts represent significant difference between compared groups at p-value < 0.05.

**Table 2:** Characteristics of fulvic acid solution of control soil and soil solutions (mean values according to depth).

Samples	Depth (cm)	n	DOC (mg.L^−1^) ± sd	pH ± sd	HIX ± sd	FI ± sd	BIX ± sd
Fulvic acid	0–30	1	–	–	15.8	1.0	0.5
	0–15	26	51.50 ± 32.24^b^	8.3 ± 0.3^c^	14.3 ± 6.5^b^	1.3 ± 0.1^a^	0.7 ± 0.8^a^
Soil solutions	15–30	20	41.55 ± 23.60^b^	8.2 ± 0.3^bc^	11.3 ± 3.8^b^	1.4 ± 0.1^b^	0.8 ± 0.6^b^
	30–60	18	32.04 ± 22.34^ab^	8.1 ± 0.2^b^	6.7 ± 3.6^a^	1.5 ± 0.1^b^	0.8 ± 0.8^c^
	60–100	14	10.55 ± 9.42^a^	7.6 ± 0.2^a^	4.8 ± 1.8^a^	1.5 ± 0.1^b^	0.9 ± 0.1^c^

Notes: The subscripts letters after the data indicate the similarity and the differences. The same alphabet indicates that the compared group are similar. Different subscripts represent significant difference between compared groups at p-value < 0.05.

Pearson's correlation coefficients (r) between various solutions for all soils depths from field systems and seasons (n = 78) are reposted in Table 3 in addition to the correlations between the variables included in PCA and the first two dimensions (Table 4).

**Table 3:** Pearson's correlation coefficients (r) between DOM for all soils management, depth and seasons (n = 78).

	BIX	DOC	FI	HIX
DOC	−0.88			
FI	0.96	−0.76		
HIX	−0.96	0.80	−0.94	
pH	−0.88	0.77	−0.75	0.87

Notes: Dissolved organic carbon (DOC) concentration, Humification index (HIX), Fluorescence index (FI) and Biological index (BIX). Pearson's correlation coefficients (*r*) significant at p-value < 0.001.

**Table 4:** Correlations between the variables included in PCA and the first two dimensions. The highest contribution of each variable is highlighted in bold characters (p < 0.002).

	HIX	FI	BIX	DOC	pH	Explained variance (%)	Cumulative (%)
PCA 1	**0.97**	**−0.94**	**−1.00**	**0.89**	**0.91**	**89.04**	89.04
PCA 2	−0.15	0.31	0.05	0.33	0.21	5.56	94.60

## 2 Experimental design, materials, and methods

### 2.1 Experimental site

The experiment was conducted in the Hydrogeological Experimental Site which is a part of the Network of National Hydrogeological Sites (SNO H+) and of the French network of Critical Zone Observatories: Research and Applications (OZCAR) of the University of Poitiers located in the Regional Observatory of Deffend (OR, Mignaloux-Beauvoir, France). The soil is a luvic cambisol fully characterized previously [5]. The experiment filed, currently under grassland, was previously divided into 3 control plots and 3 amended plots. Each plot had a total area of 3 × 3 m^2^. The plots were separated in all direction by a border space of 2 m. The compost was produced at the platform of La Villedieu du Clain (France) in open air windows from 80% green waste and 20% biowaste obtained from district selective collection.

Urban green waste compost was amended on 3 plots (T, 150 t.ha^−1^) closed to 3 control plots (C, grassland without amendment), all equipped with suction cups.

### 2.2 Collection and preparation of samples

Each plot was equipped with 12 ceramic suction cups (31 mm, SDEC, France) to collect soil solutions disposed in triplicates at 15, 30, 60 or 100 cm depth. The sampling was performed in autumn and spring. Samples were then filtered through 0.45 μm filters (Polycap 75 TF, Whatman) and the pH was measured. Samples were then stored in glass bottles at 4 °C prior to analysis.

### 2.3 Bulk analysis

Total Organic Carbon Analyzer (TOC- V CPN, Japan) was run in Non Purgeable Organic Carbon mode (NPOC). Samples for Dissolved Organic Carbon (DOC) analyses were acidified with 2 N HCl and sparged with high purity air to remove inorganic carbon. For each sample, DOC was the mean of a least three injections (standard deviation < 0.1 and variation coefficient < 2%).

For spectroscopic analysis, samples were diluted with pure water to reach a DOC content of 5 mg.L^−1^. Samples were then placed in a 1 cm quartz cuvette thermostated at 20 °C. 41 individual emission scans (300–550 nm, at 5 nm intervals) at 5 nm excitation wavelength intervals between 200 nm and 400 nm were recorded. Values were all normalized to the Raman peak of the Milli-Q water. EEM plots were assembled by combining the individual emission spectra using SigmaPlot 11 (Systat Software, Inc., San Jose, CA, USA).

All statistical tests were conducted by R Core Team (2019). Principal component analysis (PCA) and “Pearson” correlation analysis were conducted on all the leachates datasets to evaluate the correlation structure among the parameters measured. Five quantitative variables were analyzed: DOC, pH and fluorescence indexes (HIX, FI and BIX). Pearson's product moment correlation was used to determine the correlation of the different components to physical, biological and chemical environmental variables. The means for each of sampling periods were used in the correlation analysis against the five quantitative parameters. Pearson's product moment correlation coefficients were analyzed using Sigma Stat version 3.5 (Systat Software).
